# The Cell Doctrine. Its History and Present State

**Published:** 1870-04

**Authors:** 


### BIBLIOGRAPHICAL NOTICES.

*The Cell Doctrine. Its History and Present State.* By James Tyson, M.D. Philadelphia: Lindsay & Blackiston, 1870.

This volume is inscribed to the Medical Class of the University of Pennsylvania, in which institution the author is Lecturer on Microscopy. This work is one which has long been desired by both medical and dental students, as it includes views which are only to be found in numerous and expensive works, and as collected in this small volume present a convenient form for study, &c. It is illustrated by a well executed colored plate of Dr. Beale's views, showing the production of formed material from germinal matter in epithelial cells, from section through layer of epithelium covering papillæ of frog's tongue; formation of pus, tendon, cartilage, muscle, elastic, tissue and nerve; amœba and nutrition of cell; and wood cuts illustrating the globular theory, cellular tissue, formation of nuclei and cells from molecules, diagram of the investment theory, formation of pus, development of cancer, connective tissue corpuscles, and formation of elastic tissue, with a copious bibliography. The work is published in a handsome form, in large type and on good paper.

